# Induction of Hematopoietic Microchimerism by Gene-Modified BMT Elicits Antigen-Specific B and T Cell Unresponsiveness toward Gene Therapy Products

**DOI:** 10.3389/fimmu.2016.00360

**Published:** 2016-09-15

**Authors:** Jérémie Martinet, Gwladys Bourdenet, Amine Meliani, Laetitia Jean, Sahil Adriouch, Jose L. Cohen, Federico Mingozzi, Olivier Boyer

**Affiliations:** ^1^Normandie University, UNIROUEN, Pathophysiology and Biotherapy of Inflammatory and Autoimmune Diseases, INSERM, CHU Rouen, Rouen, France; ^2^U974, INSERM, University Pierre and Marie Curie, Paris, France; ^3^Genethon, Evry, France; ^4^U955 Team 21, Inserm, Créteil, France

**Keywords:** gene therapy, hemophilia B, tolerance, microchimerism, animal model

## Abstract

**Background:**

Gene therapy is a promising treatment option for hemophilia and other protein deficiencies. However, immune responses against the transgene product represent an obstacle to safe and effective gene therapy, urging for the implementation of tolerization strategies. Induction of a hematopoietic chimerism *via* bone marrow transplantation (BMT) is a potent means for inducing immunological tolerance in solid organ transplantation.

**Objectives:**

We reasoned, here, that the same viral vector could be used, first, to transduce BM cells for inducing chimerism-associated transgene-specific immune tolerance and, second, for correcting protein deficiencies by vector-mediated systemic production of the deficient coagulation factor.

**Methods:**

Evaluation of strategies to induce B and T cell tolerance was performed using *ex vivo* gene transfer with lentiviral (LV) vectors encoding coagulation factor IX (FIX) or the SIINFEKL epitope of ovalbumin. Following induction of microchimerism *via* BMT, animals were challenged with *in vivo* gene transfer with LV vectors.

**Results:**

The experimental approach prevented humoral immune response against FIX, resulting in persistence of therapeutic levels of circulating FIX, after LV-mediated gene transfer *in vivo*. In an ovalbumin model, we also demonstrated that this approach effectively tolerized the CD8^+^ T cell compartment in an antigen-specific manner.

**Conclusion:**

These results provide the proof-of-concept that inducing a microchimerism by gene-modified BMT is a powerful tool to provide transgene-specific B and T cell tolerance in a gene therapy setting.

## Introduction

A major complication of enzyme replacement therapy in hemophilia and other genetic defects is the development of immune responses toward the recombinant therapeutic protein (1–3). Similarly, a potential important concern for gene therapy is represented by the risk of immunization against the therapeutic transgene product, as shown in animal models of hemophilia B (4–8). In particular, while factor IX (FIX) gene therapy using adeno-associated or lentiviral (LV) vectors *in vivo* is a promising treatment option for hemophilia B (9, 10), humoral and cell-mediated immune responses triggered by the transgene may result in lack of therapeutic efficacy (11, 12). Only few tolerizing strategies have been investigated to tackle this issue, mostly by targeting FIX expression to the liver (5, 13) or detargeting transgene expression from antigen-presenting cells (11). Some other approaches have also been proposed to induce tolerance to factor VIII (FVIII) in hemophilia A, i.e., administration of B cell blasts transduced by FVIII-immunodominant domains using a retrovirus-mediated gene transfer (14) or intraosseous infusion of LV encoding FVIII under the control of platelet-specific promoters (15).

Induction of a hematopoietic chimerism is a potent means for inducing immunological tolerance in solid organ transplantation. For instance, transplantation of alloantigen-expressing BM cells results in a strong state of tolerance in allogeneic (16) or syngeneic gene-modified settings (17). In hemophilia, expression of coagulation factors at therapeutic levels by transduced BM cells has been shown to provide FVIII- or FIX-specific tolerance in settings where transgene expression is restricted to the hematopoietic compartment (18–22). Here, we evaluated the hypothesis that induction of a microchimerism (<0.5%) by grafting LV-modified BM may be sufficient to elicit transgene-specific tolerance and to sustain transgene expression after subsequent systemic LV administration or LV injection to an extra-hematopoietic tissue.

## Materials and Methods

### Lentiviral Vectors and Gene Transfer

**Figure 1:**
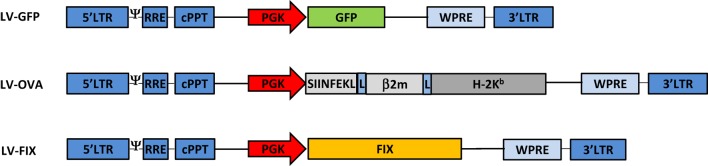
**Lentiviral vectors design**. Schematic representation of the LV-GFP, LV-OVA, and LV-FIX lentiviral vectors.

We developed LV-OVA and LV-FIX vectors by replacing the GFP gene of the PGK promoter-driven LV-GFP vector (23) by a SIINFEKL/β2-microglobulin/H-2K^b^ fusion construct (24) or human FIX cDNA (25), respectively (Figure 1). LV titers (expressed as transducing units, TU/mL) were determined by flow cytometry for LV-OVA and LV-GFP (26) and by qPCR for LV-FIX (10). BM cells from female Ly5.1 C57BL/6 (Ly5.1 B6) mice (Charles River Laboratories) were transduced with LV at a multiplicity of infection of 1 (17).

### Induction of BM Chimerism

Animal experiments were approved by an ethics committee according to French legislation (authorization N/35-11-12/58/11-15).

Ly5.2 C57BL/6 (B6) mice (Charles River Laboratories) were sub-lethally irradiated (5 Gy) using an X-ray Faxitron apparatus. BM cells from Ly5.1 B6 mice, transduced by LV and 10^7^ cells, were injected IV into irradiated recipients. Two months after injection, BM cells from transplanted mice were stained with APC-labeled anti-CD45.1 and PerCP-Cy5.5-labeled anti-CD45.2 monoclonal antibodies (eBioscience). The percentage of CD45.1^+^ donor-type among CD45.2^+^ recipient-type BM cells was determined by flow cytometry (FACS CantoII, Becton Dickinson).

In LV-OVA experiments, transduction efficacy was determined by flow cytometry after staining with the 25-D1.16 monoclonal antibody recognizing the H-2K^b^-OVA complex (eBioscience).

### Transgene Persistence

Mice received intramuscular injection of LV-OVA (4 × 10^9^ TU/mouse) or IV injection of LV-FIX (10^9^ TU/mouse). LV-OVA mRNA was quantified from injected muscles by qPCR with SYBR green (Roche) using a LightCycler480 Roche, as described (27, 28). Relative amounts of LV-Ova mRNA were determined using a standard curve (serial dilutions of plasmid) and normalized by the amount of Eef2. Alternatively, FIX production was measured in plasma by ELISA (29).

### Immune Response toward the Transgene Product

Subcutaneous injection of 20 μg human FIX (LFB, Les Ulis, France) emulsified in complete Freund’s adjuvant (Sigma) was carried out on the day of IV injection of LV-FIX to provoke immunization, as classically performed (10). The level of FIX-specific antibodies was measured in plasma by ELISA (30).

CD8^+^ T cells recognizing the OVA-specific SIINFEKL peptide were numerated by flow cytometry after staining with PE-conjugated H-2K^b^/SIINFEKL dextramers (Immudex).

## Results

**Figure 2:**
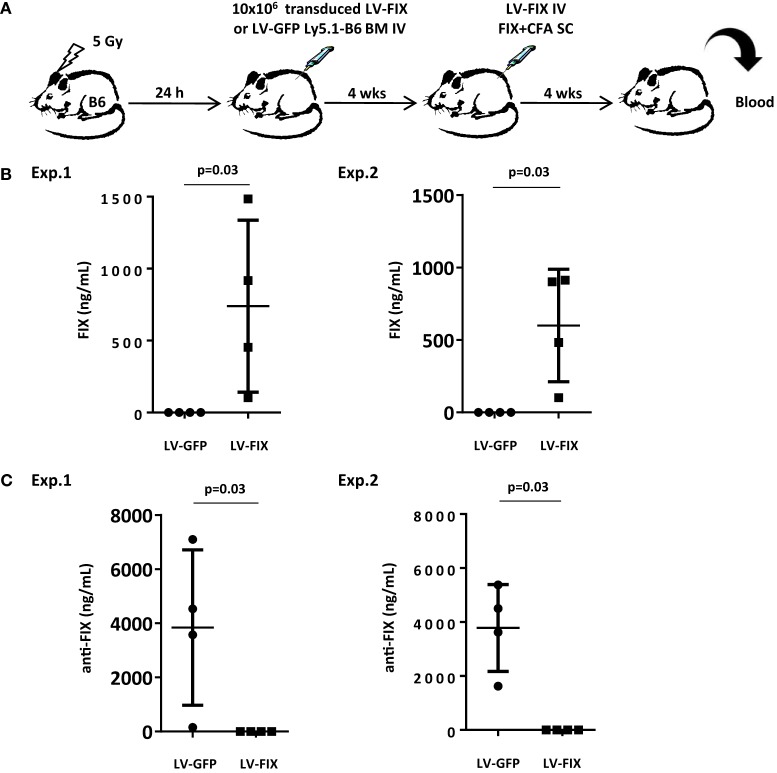
**Non-lethally irradiated mice grafted with BM cells transduced by LV-FIX are tolerized toward human FIX**. **(A)** Non-lethally irradiated (5 Gy) B6 mice (Ly5.2) were transplanted with BM cells transduced *ex vivo* with LV-FIX (or control LV-GFP) from Ly5.1 congenic B6 mice. One month after transplantation, chimeric mice were immunized by subcutaneous (SC) injection of recombinant FIX in complete Freund’s adjuvant and injected IV with the FIX-LV. **(B)** One month later, production of FIX in blood was evaluated by ELISA. Results are from two independent experiments using *N* = 4 mice per group. **(C)** Anti-FIX antibodies were titrated by ELISA.

We, first, evaluated whether inducing microchimerism by transplantation of gene-modified BM could be sufficient for inhibiting the production of transgene-specific antibodies in a gene therapy setting. For this purpose, we attempted to tolerize non-lethally irradiated Ly5.2 B6 mice against FIX by injecting BM cells from Ly5.1 congenic animals that had been transduced *ex vivo* with LV-FIX or LV-GFP as control (Figure 2A). One month after BM graft, mice were tested for human FIX expression in plasma. No circulating human FIX was found in both groups, demonstrating that the low frequency of LV-FIX expressing BM cells after non-lethal conditioning is not enough to produce circulating human FIX. Then, mice were challenged using a strong immunogenic regimen (FIX in complete Freund’s adjuvant) to induce both cell-mediated and humoral responses to FIX antigens, as described (5). At the same time, mice were injected IV with 10^9^ TU of LV-FIX to promote the endogenous expression of human FIX. One month later, we observed a mixed BM chimerism where donor Ly5.1^+^ cells represented 21 ± 2.1% of the total BM cells. In mice that had received LV-FIX-modified BM cells, there was expression of human FIX at therapeutic level in plasma (612 ± 591 ng/mL, Figure 2B). In contrast, no circulating human FIX (<1.5 ng/mL) was found in controls that had received LV-GFP BM. These results indicate that the tolerization regimen had prevented mice from mounting an anti-FIX humoral immune response. Indeed, no FIX-specific antibodies were found in tolerized mice, whereas there was a strong humoral response in controls (Figure 2C).

**Figure 3:**
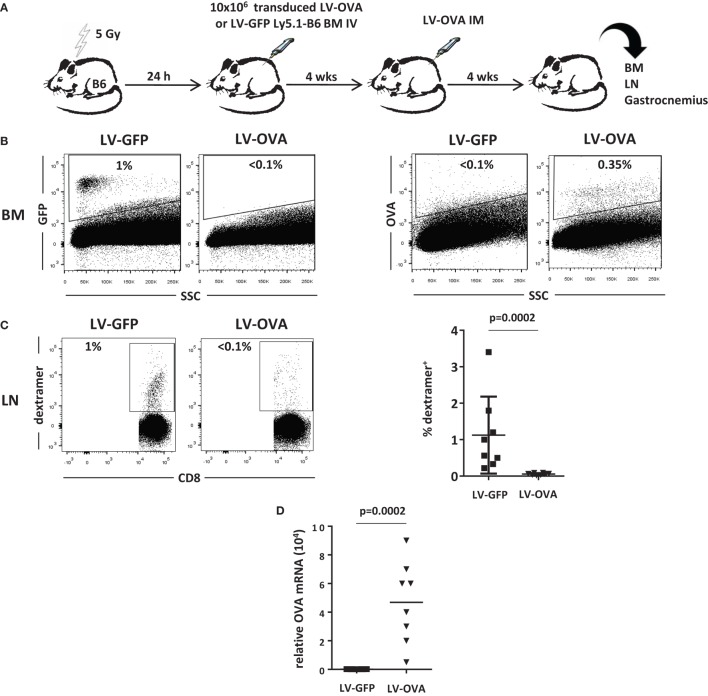
**Non-lethally irradiated mice grafted with LV-OVA transduced BM cells are tolerized toward a membrane-bound transgene product**. **(A)** Irradiated (5 Gy) B6 mice were transplanted with Ly5.1 B6 BM cells that had been transduced *ex vivo* with a LV expressing the SIINFEKL immunodominant peptide of ovalbumin (OVA257-264) covalently linked to H-2K^b^ so that to be expressed at the cell surface (LV-OVA) or a LV-GFP control. Then, chimeric mice were injected intramuscularly (IM) in one gastrocnemius with LV-OVA. **(B)** One month later, BM transgene expression was evaluated by flow cytometry for H-2Kb-OVA and GFP expression. **(C)** Immunization against transgenic OVA was evaluated in draining lymph nodes by staining specific CD8^+^ T cells with H-2Kb/SIINFEKL dextramers. Results are from two independents experiments with eight mice per group. **(D)** Expression of OVA mRNA was evaluated by RT-qPCR in injected gastrocnemius.

We, next, evaluated the effect of this strategy on the cytotoxic cellular immune response in another model where the transgene product is membrane-bound and expressed from muscle. 5-Gy-conditioned B6 recipient mice were tolerized by grafting BM cells derived from Ly5.1 congenic donor mice transduced with a LV expressing the SIINFEKL immunodominant peptide of ovalbumin (OVA_257–264_) fused to H-2K^b^ (LV-OVA) or a control LV-GFP (Figure 3A). One month post-grafting, mice from the LV-OVA and LV-GFP tolerized groups both received LV-OVA intramuscularly for studying the OVA-specific cellular response and transgene persistence.

One month after LV-OVA challenge, the mixed chimerism could be confirmed and Ly5.1 donor cells represented 20 ± 3% of BM cells. Gene-modified cells only represented a minority of BM cells after conditioning, i.e., 1.0 ± 0.6% (GFP) or 0.3 ± 0.1% (OVA) (Figure 3B).

The cellular immune response was quantified by evaluating the percentage of SIINFEKL-specific CD8^+^ T cells in lymph nodes. In control mice that had received LV-GFP BM cells, specific CD8^+^ T cells expanded to reach 1.2 ± 0.5%, whereas they remained below the level of detection in tolerized mice that had received LV-OVA BM cells (Figure 3C). Hence, induction of microchimerism using gene-modified BM cells also prevented mice from mounting a transgene-specific cellular immune response.

To evaluate whether this state of immunological unresponsiveness also supported transgene persistence, we determined the level of transgene expression in the injected muscle. In control LV-GFP-tolerized mice, there was no detectable expression, indicating that transduced cells had been rejected by the CD8^+^ cytotoxic response (Figure 3D). In contrast, mice that had been tolerized by LV-OVA BM cells still expressed significant levels of transgenic mRNA 1 month after the intramuscular LV-OVA challenge.

## Discussion

One of the major causes of FIX replacement therapy failure in hemophilia B is the development of inhibitors, i.e., anti-FIX antibodies (3). Similarly, development of antibodies in protein- and gene-replacement therapy represents an important potential complication in the treatment of many diseases (1–3). Furthermore, transgene-specific T CD8^+^ lymphocytes can destroy transduced cells and provoke failure of gene therapy as seen in preclinical animal models of hemophilia B (31, 32) and in clinical trials of gene therapy for muscular dystrophy (33).

Here, we demonstrated that expression of a transgene in a minority of BM cells after a non-lethal conditioning regimen is able to tolerize mice in an antigen-specific manner. With this intervention, we were able to prevent both humoral and CD8^+^ T cells responses, allowing for sustained transgene expression after subsequent systemic or tissue-specific *in vivo* gene transfer.

Induction of tolerance by transplantation of syngeneic BM cells that had been transduced by an alloantigen-specific transgene was previously explored (17). The underlying mechanisms involved T cell negative selection in the thymus, leading to a robust and lasting central T cell tolerance (34). Another study showed that grafting alloantigen-expressing BM cells is able to induce regulatory T cells, leading to peripheral tolerance (35). It is presumable that such mechanisms are also involved in the approach described, herein.

Oral tolerance or liver gene transfer has been used to tolerize mice to transgene products. Oral tolerance can be efficient in the context of gene therapy, but requires repeated oral administration of high doses of the tolerogen (28). Liver gene transfer is able to reduce the level of FIX-specific inhibitors and sustain long-term transgene expression (10, 13, 31, 36), but cannot be applied for other organs gene therapy. Notably, the approach proposed herein could be proposed for liver, muscle, or other organ gene transfer to improve transgene tolerance and long-term expression after a unique administration of a low number of gene-modified BM cells.

The present results are consistent with that of other studies in which FIX was expressed from transduced BM cells for both tolerization and therapeutic purposes. However, a high level of transduction was required to achieve tolerance and to also produce therapeutic levels of FIX (18–22). Here, we show that, with the current strategy, only a low frequency of LV-modified BM cells (less than 0.5%) is sufficient to tolerize mice. Importantly, this approach is compatible with different gene therapy settings, i.e., injecting the vector in blood, muscle, or potentially other extra-hematopoietic tissues. Therefore, it may prove useful for providing transgene-specific tolerance in the context of gene therapy of monogenic diseases beyond hemophilia.

In conclusion, the present results provide proof-of-concept of induction of tolerance *via* syngeneic microchimerism in a clinically translatable gene therapy setting. The use of LV-transduced BM cells after reduced intensity conditioning (37, 38) may represent a feasible candidate approach for augmenting the probability of success of different indications of gene therapy, by preventing detrimental humoral and cellular immune responses to the therapeutic transgene whatever its mode of expression.

## Author Contributions

JM, GB, AM, LJ, and SA performed experiments; JM, JC, FM, and OB designed the research, analyzed data, and wrote the paper.

## Conflict of Interest Statement

The authors declare that the research was conducted in the absence of any commercial or financial relationships that could be construed as a potential conflict of interest.
